# Rotation-Sensitive Feature Enhancement Network for Oriented Object Detection in Remote Sensing Images

**DOI:** 10.3390/s26020381

**Published:** 2026-01-07

**Authors:** Jiaxin Xu, Hua Huo, Shilu Kang, Aokun Mei, Chen Zhang

**Affiliations:** College of Information Engineering and Artificial Intelligence, Henan University of Science and Technology, Luoyang 471000, China; xujiaxin0523@163.com (J.X.); ksl_life@163.com (S.K.); 240410040061@stu.haust.edu.cn (A.M.); 250410040094@stu.haust.edu.cn (C.Z.)

**Keywords:** remote sensing, feature pyramid, rotation-sensitive, attention mechanism, geometric consistency

## Abstract

Oriented object detection in remote sensing images remains a challenging task due to arbitrary target rotations, extreme scale variations, and complex backgrounds. However, current rotated detectors still face several limitations: insufficient orientation-sensitive feature representation, feature misalignment for rotated proposals, and unstable optimization of rotation parameters. To address these issues, this paper proposes an enhanced Rotation-Sensitive Feature Pyramid Network (RSFPN) framework. Building upon the effective Oriented R-CNN paradigm, we introduce three novel core components: (1) a Dynamic Adaptive Feature Pyramid Network (DAFPN) that enables bidirectional multi-scale feature fusion through semantic-guided upsampling and structure-enhanced downsampling paths; (2) an Angle-Aware Collaborative Attention (AACA) module that incorporates orientation priors to guide feature refinement; (3) a Geometrically Consistent Multi-Task Loss (GC-MTL) that unifies the regression of rotation parameters with periodic smoothing and adaptive weight mechanisms. Comprehensive experiments on the DOTA-v1.0 and HRSC2016 benchmarks show that our RSFPN achieves superior performance. It attains a state-of-the-art mAP of 77.42% on DOTA-v1.0 and 91.85% on HRSC2016, while maintaining efficient inference at 14.5 FPS, demonstrating a favorable accuracy-efficiency trade-off. Visual analysis confirms that our method produces concentrated, rotation-aware feature responses and effectively suppresses background interference. The proposed approach provides a robust solution for detecting multi-oriented objects in high-resolution remote sensing imagery, with significant practical value for urban planning, environmental monitoring, and security applications.

## 1. Introduction

Advances in Earth observation technologies have enabled high-resolution satellites (e.g., WorldView, GF-2) and UAV platforms to capture global imagery with unprecedented spatial and temporal resolution. These datasets support critical applications, including smart city management [1], precision agriculture [2], disaster monitoring [3], maritime protection [4], and military reconnaissance [5]. Object detection, a core technique for intelligent remote sensing interpretation, aims to automatically localize and classify objects such as vehicles, ships, aircraft, and buildings [6]. While deep learning detectors like Faster R-CNN [7] and YOLO [8,9] have excelled on natural images, their direct application to remote sensing imagery remains challenging due to the “bird’s-eye view” perspective: objects often appear densely distributed with arbitrary orientations, complex backgrounds, and widely varying scales [10,11,12,13]. Using conventional horizontal bounding boxes (HBBs) to represent such objects introduces irrelevant background, increases overlap in dense regions, and degrades both localization and classification accuracy [14,15].

To address the limitations of horizontal bounding boxes, rotated object detection (ROD) employs oriented rectangles to tightly fit object contours and orientations [16,17,18,19]. In recent years, the field has evolved along two main paradigms. Single-stage detectors (e.g., R^3^Det [17], S^2^ANet [20]) prioritize efficiency by directly regressing rotated parameters, while two-stage detectors (e.g., RoI Transformer [21], Oriented R-CNN [19]) achieve higher accuracy through oriented region proposal and feature alignment mechanisms. Despite significant progress on benchmarks like DOTA [10], applying these detectors to complex remote sensing imagery still faces three fundamental challenges, which we aim to address systematically.

**First, the feature representation challenge.** Remote sensing imagery exhibits extreme scale variations and complex backgrounds, requiring detectors to possess multi-scale fusion and orientation-sensitive feature enhancement capabilities. While feature pyramid networks (FPN) [22] and variants like PANet [23] and BiFPN [24] improve semantic consistency, their axis-aligned convolutional kernels cannot adapt to the geometric deformations of rotated objects. This results in insufficient orientation sensitivity [18], geometric structure degradation [25], and multi-scale mismatch [26], collectively limiting accurate feature representation.

**Second, the challenge of geometric alignment.** The arbitrary orientations of rotated objects cause standard RoIAlign to misalign with the target geometry during feature extraction. Existing approaches, such as RoI Transformer [21], R2CNN [27], and Gliding Vertex [28], attempt geometric correction but often introduce high computational costs or remain sensitive to background noise in cluttered scenes. Enhancing directional consistency and feature focus efficiently remains a key challenge.

**Third, the challenge of optimization.** The parameter representation of rotated boxes (e.g., (x,y,w,h,θ)) is more complex. Decoupled regression with losses like Smooth L1 [29] often leads to inconsistent gradients, unstable convergence [30], and a heavy reliance on manual weight tuning. Losses based on approximate rotational IoU (e.g., PIoU [31]) or Gaussian modeling (e.g., KLD [32], GWD [33]) have demonstrated improved sensitivity and continuity. However, they still face approximation errors under extreme aspect ratios or efficiency issues with small objects. Consequently, developing a loss function that can jointly optimize all geometric parameters with stable gradients in complex scenes remains a central challenge.

To address these interrelated challenges, we propose a key insight: if we can closely integrate rotation sensitive feature representation and geometric constraints in the whole detection process, we can construct a unified detection framework, which can significantly improve the accuracy and robustness of detection. Specifically, we believe that synergistically improving the ability of multi-scale feature learning, achieving accurate geometric alignment of the rotated regions, and stably optimizing the rotation parameters are three complementary keys. To this end, we propose a Rotation Sensitive Feature Pyramid Network (RSFPN)—an end-to-end trainable detection framework that aims to implement the above ideas holistically. RSFPN contains three core modules of collaborative design: Dynamic adaptive Feature Pyramid Network (DAFPN), which maintains cross-scale geometric consistency through bidirectional feature fusion. The Angle-Aware Collaborative Attention (AACA) module guided the feature enhancement along the main axis of the object according to the direction information, and Geometric Consistency Multi-Task Loss (GC-MTL), which unifies rotational parameter regression into a stable optimization objective. These modules work together so that the network remains sensitive to rotation from feature extraction to result output. We conduct comprehensive experiments on DOTA-v1.0 [10] and HRSC2016 [13] benchmarks to rigorously verify the validity of the above ideas and the performance of the RSFPN framework.

## 2. Related Work

### 2.1. Feature Pyramids and Multi-Scale Fusion

Feature Pyramid Networks (FPN) [22] remain fundamental for handling scale variation. Various extensions—including PANet [23], BiFPN [24], NAS-FPN [26], and ASFF [34]—enhance cross-scale fusion through more efficient or adaptive pathways. In remote sensing, models such as DRBox [35] and R-DFPN [36] incorporate multi-scale structures tailored for rotated objects.

However, existing approaches often overlook the preservation of fine-grained geometric cues essential for rotation-sensitive targets. Motivated by PANet and BiFPN, our DAFPN introduces semantically guided dynamic upsampling [37] and structure-preserving downsampling, enabling more precise bidirectional fusion and improved multi-scale geometric representations.

Beyond dedicated feature pyramid designs, mainstream multi-scale detection employs alternative paradigms. Single-stage detectors like RetinaNet [38] and its rotated variants [17] rely on dense anchor sampling across pyramid levels, while anchor-free methods such as FCOS [39] and CenterNet [40] assign scales based on point or center predictions. These methods often rely on heuristic scale assignment and may not explicitly preserve geometric consistency between feature levels -this is the core motivation for our structured bidirectional fusion of DAFPN.

### 2.2. Attention Mechanisms in Object Detection

Attention mechanisms adaptively reweight features, enabling networks to focus on target-relevant information while suppressing background noise. Channel attention was first introduced by SENet [41], and CBAM [42] sequentially integrates channel and spatial attention for a lightweight general-purpose structure. GCNet [43] and Non-Local Networks [44] capture long-range dependencies, while Transformers [45] have inspired detectors like DETR [46] to model global relationships effectively.

Nevertheless, these methods largely ignore explicit geometric priors such as object orientation, limiting their effectiveness in rotated detection. To address this gap, our AACA module generates angle-guided direction-aware attention maps, enabling collaborative enhancement of spatial and channel responses along target-aligned axes.

To enhance features for rotated objects, common strategies include: rotation-equivariant networks (e.g., ReDet [47]) that build in symmetry; deformable convolutions [48] that adapt sampling grids; explicit feature alignment modules like RoI Transformer [21] and Rotated RoIAlign [19]. In contrast, our AACA module adopts a complementary, lightweight attention paradigm that directly injects the predicted orientation as a spatial-channel prior, enhancing discriminability without altering the core feature alignment process.

### 2.3. Loss Functions for Rotated Bounding Boxes

Rotated bounding boxes are typically represented by either the eight-point polygon format or the five-parameter (x,y,w,h,θ) formulation, and their regression introduces challenges such as angle discontinuity and parameter coupling. Prior works attempt to address these issues via improved losses or encodings, such as IoU-Smooth L1 in SCRDet [30], CSL’s [49] circular label smoothing, DCL’s dense angle encoding [50], and Gaussian-based metrics including GWD and KLD [32,33]. PSC [51] further stabilizes angle regression through phase-based modeling.

However, most existing approaches optimize center, scale, and angle parameters independently with manually tuned loss weights, often resulting in unstable gradients and inconsistent convergence [17,30]. To overcome these limitations, our GC-MTL unifies all rotated box parameters into a geometrically consistent multi-task framework with angle-period smoothing and adaptive weighting to improve optimization stability.

Alongside advanced loss functions, alternative regression paradigms have emerged. Gaussian modeling methods (e.g., KLD [32], GWD [33]) represent boxes as distributions for differentiable loss. Another line of work uses progressive refinement [20] or repoints [18] to decouple optimization. While effective, they can add complexity. Our GC-MTL differentiates itself by offering a unified, parameter-efficient loss that enforces geometric consistency among all parameters within a standard regression head, ensuring stable training without architectural modification.

### 2.4. Comparative Analysis

To clearly position our work and highlight its systematic advancements, Table 1 contrasts the technical designs and inherent limitations of key representative methods. Unlike prior works that address these challenges in isolation, our RSFPN framework provides a unified solution: it simultaneously enhances rotation-sensitive feature representation (via DAFPN), geometric alignment and focus (via AACA), and regression stability (via GC-MTL). The synergistic effect of these modules, as evidenced by our ablation studies, is what enables RSFPN to effectively overcome the combined limitations outlined in the table.

## 3. Methods

### 3.1. Overall Framework

**Figure 1:**
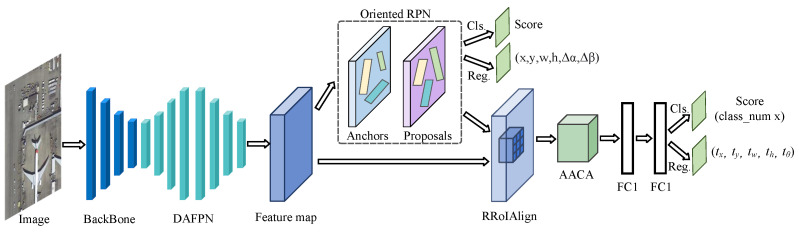
The overall architecture of the proposed Rotation-Sensitive Feature Pyramid Network (RSFPN).

We propose a novel Rotation-Sensitive Feature Pyramid Network (RSFPN) to effectively address the challenges of rotated object detection in remote sensing imagery. As shown in Figure 1, RSFPN adopts a hierarchical and modular design that decomposes the detection pipeline into three core components: the feature extraction and enhancement module, the rotated proposal generation and alignment module, and the feature refinement and detection module. This structured design not only improves the interpretability of the network architecture but also strengthens the synergistic interactions among modules.

The input image first passes through a ResNet-50 backbone to extract fundamental feature representations. These features are then fed into the Dynamic Adaptive Feature Pyramid Network (DAFPN). Unlike conventional feature pyramids, DAFPN incorporates a semantics-guided upsampling path and a structure-enhanced downsampling path to achieve bidirectional interaction and deep fusion across multiple feature levels, thereby providing high-quality and multi-scale feature representations for subsequent processing. In the rotated proposal generation and alignment module, the Oriented Region Proposal Network (Oriented RPN) takes the enhanced features produced by DAFPN and efficiently generates a set of rotation-aligned proposals using a midpoint-offset parameterization. Each proposal is subsequently processed by the Rotated RoIAlign operation, which extracts geometrically aligned features from the corresponding feature maps and effectively mitigates feature misalignment issues commonly encountered in rotated object detection. In the feature refinement and detection module, the aligned features obtained via Rotated RoIAlign are forwarded to the Angle-Aware Cooperative Attention (AACA) module. This module treats the rotation angle as a strong geometric prior and generates a direction-aware mask through a lightweight mapping network. The mask guides the cooperative interaction of spatial and channel attention mechanisms, thereby enhancing discriminative target features while suppressing background noise in cluttered scenes. Finally, the AACA-enhanced features are sent to the detection head for classification and refined bounding-box regression.

The entire network is trained in an end-to-end manner and is jointly optimized using the proposed Geometrically Consistent Multi-Task Loss (GC-MTL). This loss function unifies the regression of all rotated bounding box parameters within a single geometrically consistent framework. By incorporating angle-period handling, shape constraints, and an adaptive weighting mechanism, GC-MTL ensures stable training and reliable convergence, directly addressing the core optimization challenges in rotated object detection.

### 3.2. Feature Extraction and Enhancement Module

#### 3.2.1. Backbone Feature Extraction

**Figure 2:**
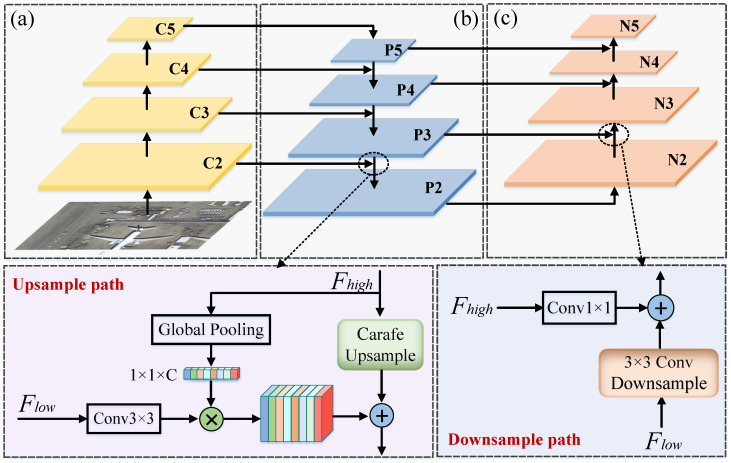
Overall architecture of the proposed Dual-Path Adaptive Feature Pyramid Network (DAFPN): (**a**) Backbone; (**b**) Semantic-guided upsample path; (**c**) Structure-enhanced downsample path.

We employ a ResNet-50 backbone pretrained on the ImageNet dataset to extract four hierarchical feature maps, denoted as {C2,C3,C4,C5} (see Figure 2), from the input image. These feature maps have spatial downsampling ratios of 4×, 8×, 16×, and 32×, respectively. They provide the foundation for multi-scale feature representation, with gradually decreasing spatial resolution and progressively increasing channel capacity. The shallow feature maps (e.g., C2 and C3) retain abundant spatial details, which are beneficial for localizing small objects, while the deeper feature maps (e.g., C4 and C5) contain richer semantic information, which is advantageous for object classification.

#### 3.2.2. Dynamic Adaptive Feature Pyramid Network

To enhance the multi-scale representation capability required for complex objects in remote sensing imagery, we propose a Dynamic Adaptive Feature Pyramid Network (DAFPN). Unlike conventional FPNs that simply perform a top-down lateral aggregation, DAFPN adopts a bidirectional feature fusion architecture composed of a semantically guided upsampling path and a structurally enhanced downsampling path. This design enables a deep coupling between spatial details and semantic information across scales. The overall architecture is illustrated in Figure 2, where the left branch represents the upsampling path and the right branch denotes the downsampling enhancement pathway.

The upsampling path enables the effective propagation of high-level semantic information to lower layers (corresponding to the left branch in Figure 2). Specifically, the high-level feature map $F_{\mathrm{high}}$ first undergoes global average pooling to extract global contextual information, producing a channel vector $F_{\mathrm{global}}$. Meanwhile, the low-level feature map $F_{\mathrm{low}}$ is processed by a 3×3 convolution to capture local edge and texture details. The resulting feature is then modulated by $F_{\mathrm{global}}$ through channel-wise multiplication, achieving semantically guided adaptive weighting and enhancing the sensitivity of low-level features to high-level semantics. Subsequently, the high-level feature is upsampled using the CARAFE module, which generates content-aware dynamic kernels to better recover structural boundaries and alleviate aliasing artifacts commonly observed in conventional upsampling. Finally, the enhanced high-level feature and the adaptively weighted low-level feature are fused via element-wise addition to produce direction-sensitive feature maps. This guided upsampling procedure is applied recursively across all pyramid levels (C5–C2), forming a multi-scale set of direction-sensitive features {P5,P4,P3,P2}. The design substantially strengthens the ability of shallow layers to interpret high-level contextual information, thereby improving the representation capability for small objects and arbitrarily oriented targets in remote sensing imagery.

The downsampling path is designed to supplement the spatial structural information of high-level features (corresponding to the right side of Figure 2). This module begins with the low-level features produced by the upsampling path (e.g., P2) and progressively propagates spatial details upward. First, a 3×3 convolution with a step size of 2 is used to down-sample the low-level features. While this operation reduces the spatial resolution, it also expands the receptive field, which is able to capture richer spatial context information. Subsequently, a 1×1 convolution is applied to the high-level semantic features for channel alignment, followed by element-wise addition with the downsampled low-level features to achieve scale-consistent semantic–structural fusion. Through this design, high-level semantic information is stably transmitted during the downsampling process while avoiding additional spatial misalignment. The final multi-scale feature set {N2,N3,N4,N5} integrates the semantic and structural information from both the upsampling and downsampling paths, providing stronger capabilities for object localization and shape characterization.

Overall, the Dynamic Adaptive Feature Pyramid Network (DAFPN) achieves bidirectional feature fusion through semantic guidance in the upsampling path and structural compensation in the downsampling path. The upsampling path enhances the semantic representations of low-level features, while the downsampling path strengthens the spatial structure of high-level features. Through the coordinated interaction of these two paths across multiple scales, the network gains improved rotation awareness and geometric modeling capability. This design effectively boosts the detection accuracy and robustness for small objects and dense rotated targets in complex remote sensing scenes.

### 3.3. Rotated Proposal Generation and Alignment

#### 3.3.1. Oriented Region Proposal Network

**Figure 3:**
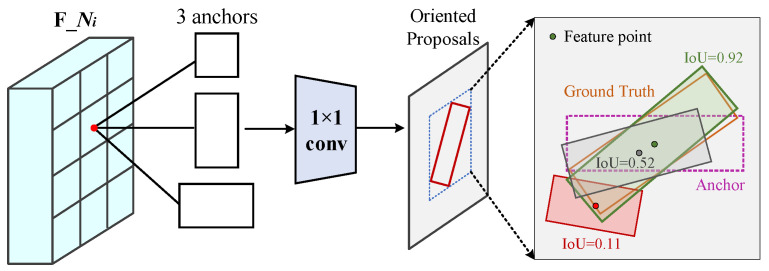
Illustration of the working mechanism of the Rotated Region Proposal Network (R-RPN). This figure depicts the core workflow of the R-RPN module. The enlarged panel on the right presents the sample assignment strategy during training.

Following the oriented region proposal design in Oriented R-CNN, our framework employs a lightweight fully convolutional network to generate high-quality rotated proposals (see Figure 3). The module takes the multi-scale feature maps enhanced by DAFPN, namely $\left\{N_{2},N_{3},N_{4},N_{5}\right\}$, as input. Each feature level corresponds to a different receptive field, enabling the network to accommodate targets of varying scales. At each feature level, a 3×3 convolution layer is first applied to transform the input features and produce a 256-dimensional shared feature representation. This shared representation is then fed into two parallel 1×1 convolutional branches. The classification branch estimates the objectness score at each spatial location, while the regression branch predicts the geometric parameters of the rotated bounding boxes.

At each spatial location of the feature maps, three horizontal anchor boxes are predefined with aspect ratios of {1:2, 1:1, 2:1}, which correspond to slender objects (e.g., ships), approximately square objects (e.g., oil tanks), and wide rectangular objects (e.g., sports fields), respectively. The base scales of the anchors are dynamically assigned according to the feature pyramid hierarchy, specifically $\left\{N_{2}:32^{2},N_{3}:64^{2},N_{4}:128^{2},N_{5}:256^{2}\right\}$ pixels. The regression branch outputs a 6-dimensional offset vector $\left(\delta_{x},\delta_{y},\delta_{w},\delta_{h},\delta_{\alpha },\delta_{\beta }\right)$, which is decoded into the six-parameter representation of a rotated proposal box through the following transformation:

(1) $$\begin{array}{cc} w=a_{w}\cdot e^{\delta_{w}}, & \;\;\;h=a_{h}\cdot e^{\delta_{h}}\\ x=\delta_{x}\cdot a_{w}+a_{x}, & \;\;\;y=\delta_{y}\cdot a_{h}+a_{y}\\ \Delta \alpha =\delta_{\alpha }\cdot w, & \;\;\;\Delta \beta =\delta_{\beta }\cdot h \end{array}$$

The variables $\left(a_{x},a_{y},a_{w},a_{h}\right)$ denote the parameters of the anchor box, while (x,y,w,h,Δα,Δβ) represent the predicted parameters of the rotated proposal. This midpoint-offset parameterization inherits the regression formulation used for horizontal bounding boxes, while the additional terms Δα and Δβ capture the rotational characteristics of the target (see Table 2 for a notation summary). In this way, the rotational prediction is performed under a stable and bounded constraint, leading to more reliable modeling of orientation variations.

During training, we adopt strict rotated IoU thresholds to assign positive and negative samples by matching each anchor box with the corresponding ground-truth box. Specifically, an anchor box is regarded as a positive sample only when the rotated IoU ≥0.7; it is treated as a negative sample when the rotated IoU ≤0.3; Anchor boxes whose rotated IoU falls between 0.3 and 0.7 are marked as ignore samples. This strategy ensures high-quality sample selection while preventing ambiguous samples from misleading the training process.

#### 3.3.2. Rotated RoIAlign Feature Extraction

**Figure 4:**
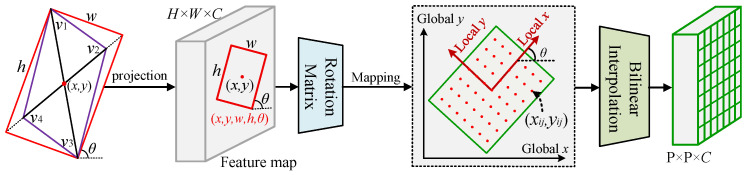
The complete processing pipeline of Rotated RoIAlign: the input feature map (H×W×C) is transformed by a rotation matrix to map the proposal coordinates into the global coordinate system, and bilinear interpolation is then applied to generate a fixed-size feature block (P×P×C).

To extract orientation-consistent features from rotated proposals, our framework employs the Rotated RoIAlign operation (see Figure 4). This operation resolves feature misalignment through precise geometric transformations and serves as a crucial step to ensure that the subsequent angle-aware collaborative attention module receives high-quality feature inputs. Given the six-parameter representation of a rotated proposal (x,y,w,h,Δα,Δβ), the four vertex coordinates are first computed based on the midpoint offset formulation:

(2) $$\begin{array}{cc} \; & v_{1}=\left(x,\;y-h/2\right)+\left(\Delta \alpha ,\;0\right)\\ \; & v_{2}=\left(x+w/2,\;y\right)+\left(0,\;\Delta \beta \right)\;\\ \; & v_{3}=\left(x,\;y+h/2\right)+\left(-\Delta \alpha ,\;0\right)\;\\ \; & v_{4}=\left(x-w/2,\;y\right)+\left(0,\;-\Delta \beta \right) \end{array}$$

Subsequently, the parallelogram proposal is rectified into an oriented rectangle to obtain the standard rotated representation (x,y,w,h,θ), where θ∈[−π/2,π/2] denotes the rotation angle defined as the inclination between the long side of the rectangle and the horizontal axis. In the local coordinate system of the rotated box, a regular P×P sampling grid (typically P=7) is constructed. The local coordinates $\left(r_{i},s_{j}\right)$ of each grid point (i,j) are computed as follows:

(3) $$r_{i}=\left(i+0.5\right)\times w/P\;-w/2\;,\;\;\;\;s_{j}=\left(j+0.5\right)\times h/P\;-h/2\;\;$$

where i,j=0,1,…,P−1. The local coordinates are then mapped to the global feature space through a rotation matrix:

(4) $$\left[\begin{array}{c} x_{ij}\\ y_{ij} \end{array}\right]=\left[\begin{array}{cc} \cos\theta & -\sin\theta \\ \sin\theta & \cos\theta \end{array}\right]\left[\begin{array}{c} r_{i}\cdot w\\ s_{j}\cdot h \end{array}\right]+\left[\begin{array}{c} x\\ y \end{array}\right]$$

Since the transformed sampling coordinates $\left(x_{ij},y_{ij}\right)$ are generally fractional, bilinear interpolation is employed to obtain continuous feature representations. For each sampling location, the four nearest integer-coordinate points on the feature map, $Q_{11},Q_{12},Q_{21},Q_{22}$, are identified, and the interpolation weights are computed based on the relative offsets Δx and Δy:

(5) $$\begin{array}{c} f\left(x,y\right)=\left(1-\Delta x\right)\left(1-\Delta y\right)\cdot f\left(Q_{11}\right)+\left(1-\Delta x\right)\Delta y\cdot f\left(Q_{12}\right)+\Delta x\left(1-\Delta y\right)\cdot f\left(Q_{21}\right)+\Delta x\Delta y\cdot f\left(Q_{22}\right) \end{array}$$

This operation ensures that smooth and continuous feature representations are obtained even when the sampling locations fall between pixels, effectively avoiding the feature distortion commonly caused by orientation inconsistencies in conventional methods. The resulting P×P×C feature tensor provides a geometrically aligned and direction-consistent input for the subsequent Angle-Aware Synergistic Attention module. This step plays a crucial role in dense small-object detection on DOTA-v1.0 and in recognizing extremely high–aspect-ratio targets on HRSC2016.

### 3.4. Feature Refinement and Detection Module

#### 3.4.1. Angle-Aware Collaborative Attention

**Figure 5:**
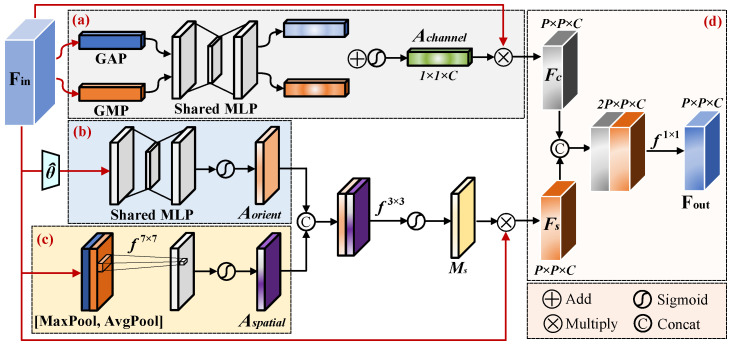
**Structure of the Angle-Aware Collaborative Attention (AACA) module.** (**a**) Channel attention branch $A_{\mathrm{channel}}$; (**b**) Angle attention branch $A_{\mathrm{orient}}$; (**c**) Spatial attention branch $A_{\mathrm{spatial}}$; (**d**) Process of feature fusion and enhancement.

To address the feature degradation of rotated objects under complex backgrounds and significant orientation variations in remote sensing imagery, we propose an Angle-Aware Collaborative Attention (AACA) module. The module takes as input the geometrically aligned features $F_{\mathrm{in}}$ produced by Rotated RoIAlign, together with their corresponding rotation angle θ, and achieves direction-aware feature enhancement through a collaborative modeling strategy across the angle, spatial, and channel dimensions. As illustrated in Figure 5, AACA consists of three parallel branches—angle attention, spatial attention, and channel attention—whose outputs are lightly fused to produce the final enhanced RoI features.

To generate the direction-aware mask $A_{\mathrm{orient}}$, the rotation angle θ is first encoded and concatenated with the normalized spatial coordinates of each position in the P×P grid. This joint representation is then processed by a two-layer MLP to produce the final mask.

(6) $$A_{\mathrm{orient}}=\mathrm{Sigmoid}\left(W_{2}\cdot \mathrm{ReLU}\left(W_{1}\hat{\theta }+b_{1}\right)+b_{2}\right)\in R^{1\times P\times P}$$

where $W_{1},b_{1},W_{2},b_{2}$ are learnable parameters, and $\hat{\theta }$ represents a feature vector that combines Angle and position information. The intermediate layer employs the ReLU activation, while the output layer uses the Sigmoid function. This mask provides a spatial prior for the main orientation of the object, complementing the traditional attention mechanisms that lack explicit directional constraints.

To capture local structural cues within the RoI, the input feature $F_{\mathrm{in}}$ is first aggregated along the channel dimension using average pooling and max pooling, producing two spatial response maps. These maps are concatenated and passed through a 7×7 convolution, followed by a Sigmoid function, to generate the spatial attention $A_{\mathrm{spatial}}$.

(7) $$A_{\mathrm{spatial}}=\mathrm{Sigmoid}\left(f_{7\times 7}\left([\mathrm{AvgPool}\left(F_{\mathrm{in}}\right)⊕\mathrm{MaxPool}\left(F_{\mathrm{in}}\right)]\right)\right)\in R^{1\times P\times P}$$

Here, ⊕ indicates channel concatenation. To model global semantic dependencies, channel statistics are extracted via global average pooling and max pooling, which are then input into a shared two-layer MLP to obtain channel-wise weights $A_{\mathrm{channel}}$.

(8) $$A_{\mathrm{channel}}=\mathrm{Sigmoid}(\mathrm{MLP}\left(\mathrm{GAP}\left(F_{\mathrm{in}}\right)+\mathrm{GMP}\left(F_{\mathrm{in}}\right))\right)\in R^{1\times 1\times C}$$

The orientation mask and spatial attention are concatenated along the channel dimension and fused using a 3×3 convolution to produce the final direction-aware spatial modulation mask $M_{s}$. This mask implements the core design of “orientation prior-guided spatial attention,” enabling spatial focus that aligns with the primary axis of the object.

(9) $$M_{s}=\mathrm{Sigmoid}(f_{3\times 3}\left[A_{\mathrm{orient}}⊕A_{\mathrm{spatial}}]\right)\in R^{1\times P\times P}$$

The input features are modulated separately by the channel attention and direction-aware spatial mask, then concatenated and fused via a 1×1 convolution to produce the final output.

(10) $$F_{\mathrm{out}}=f_{1\times 1}\left(F_{\mathrm{in}}⊙A_{\mathrm{channel}}⊕F_{\mathrm{in}}⊙M_{s}\right)\in R^{P\times P\times C}$$

Here, ⊙ denotes element-wise multiplication (utilizing the broadcasting mechanism). The AACA structure is lightweight and can be seamlessly integrated into two-stage rotated detection frameworks, significantly enhancing the discriminative capability of RoI features in complex remote sensing scenarios.

#### 3.4.2. Detection Head

Receiving the enhanced RoI features output by the Angle-Aware Collaborative Attention (AACA) module, with dimensions P×P×C, the framework employs a lightweight detection head to perform the final classification and localization tasks. The detection head consists of two main components: a shared base feature encoder and two separate branches dedicated to classification and regression, respectively.

First, the RoI features of dimensions P×P×C are flattened into a feature vector and fed into two fully connected layers (e.g., FC1 and FC2, each with a dimension of 1024). These fully connected layers serve as a shared backbone, responsible for further encoding the high-dimensional features into more discriminative representations, thereby providing an informative basis for the subsequent task-specific branches.

The classification branch receives the aforementioned encoded features and outputs a probability distribution over (K+1) classes for each candidate region through a fully connected layer, where *K* denotes the number of object categories (e.g., 15 in DOTA-v1.0), and the additional class represents the background. The regression branch shares the same input features as the classification branch and outputs, via another fully connected layer, the bounding box refinement parameters corresponding to the *K* object categories for each candidate region. This framework follows the midpoint offset representation used in Oriented R-CNN; thus, for each category, the regression branch predicts a 6-dimensional vector $\left(\delta_{x}^{\prime },\delta_{y}^{\prime },\delta_{w}^{\prime },\delta_{h}^{\prime },\delta_{\alpha }^{\prime },\delta_{\beta }^{\prime }\right)$. These parameters are employed to refine the initial proposal boxes generated by the rotated region proposal network, yielding the final precise rotated detection boxes.

#### 3.4.3. Loss Function

In rotated object detection, a bounding box is represented as (x,y,w,h,θ), where (x,y) denotes the center coordinates, *w* and *h* represent the width and height, and θ is the rotation angle, ranging within [−π/2,π/2). Conventional rotated object detection methods typically employ multiple independent loss functions to optimize these parameters, which may lead to training instability and difficulties in hyperparameter tuning. To address these issues, we propose a Geometric Consistency Multi-Task Loss (GC-MTL), which unifies the regression of all rotated bounding box parameters within a single loss function, incorporates angle periodicity handling and shape constraints, and employs an adaptive weighting mechanism to balance the contributions of different loss components. The overall loss function consists of two parts: a classification loss and a regression loss.

(11) $$L_{total}=\lambda_{cls}L_{cls}+\lambda_{reg}L_{reg}$$

Here, $L_{\mathrm{cls}}$ denotes the classification loss, for which Focal Loss [38] is employed to address class imbalance; $L_{\mathrm{reg}}$ denotes the regression loss, which jointly optimizes all parameters of the rotated bounding box; $\lambda_{\mathrm{reg}}$ represents the weighting coefficient for the regression loss, with a default value of 1.0.

(12) $$L_{cls}=-\frac{1}{N}\sum_{i=1}^{N}\sum_{c=1}^{C}\alpha_{c}{\left(1-p_{i,c}\right)}^{\gamma }y_{i,c}log\left(p_{i,c}\right)$$

Here, *N* denotes the total number of samples in a batch, *C* represents the total number of classes, $p_{i,c}$ is the predicted probability of the *i*-th sample belonging to class *c*, and $y_{i,c}$ is the ground-truth label of the *i*-th sample (one-hot encoded). $\alpha_{c}$ is the class weight factor for class *c*, used to address class imbalance, with a default value of 0.25. γ is the focusing parameter, employed to reduce the weight of easily classified samples, with a default value of 2.0.

The primary innovation proposed in this work lies in unifying the regression of all geometric parameters of rotated bounding boxes within a single loss function:

(13) $$L_{reg}=\frac{1}{N_{pos}}\sum_{i=1}^{N_{pos}}\left[L_{base}\left(i\right)+\lambda_{\theta }L_{\theta }\left(i\right)+\lambda_{shape}L_{shape}\left(i\right)\right]$$

Here, $N_{\mathrm{pos}}$ denotes the number of positive samples; $L_{\mathrm{base}}^{\left(i\right)}$ is the basic regression loss; $L_{\theta }^{\left(i\right)}$ is the angle regression loss; and $L_{\mathrm{shape}}^{\left(i\right)}$ represents the shape constraint loss. $\lambda_{\mathrm{base}}$, $\lambda_{\theta }$, and $\lambda_{\mathrm{shape}}$ are the corresponding weighting coefficients.

The basic regression loss employs the Smooth L1 loss to optimize the center coordinates and the scale parameters of the bounding box:

(14) $$L_{base}\left(i\right)=\sum_{j\in \{x,y,w,h\}}smooth_{L1}\left(\Delta_{ij}\right)$$

(15) $${\mathrm{Smooth}}_{L_{1}}\left(x\right)=\left\{\begin{array}{cc} 0.5x^{2}/\beta & \mathrm{if}\;|x|<\beta \\ |x|-0.5\beta & \mathrm{otherwise} \end{array}\right.$$

The term $\Delta_{ij}$ denotes the difference between the predicted value and the ground truth, with the smoothing coefficient set to β=1.0 by default. This loss applies a quadratic penalty to small errors and a linear penalty to large errors, effectively balancing convergence speed and training stability.

To address the half-period property of the rotation angle θ∈[−π/2,π/2), we design an angle regression loss that maps the angular discrepancy into a normalized range through the arctan function, while automatically handling the periodicity issue:

(16) $$L_{\theta }\left(i\right)=\frac{2}{\pi }\cdot \arctan\left(\frac{\left|\left.p_{\theta_{i}}-g_{\theta_{i}}\right|\right.}{\varepsilon }\right)$$

where $p_{\theta_{i}}$ and $g_{\theta_{i}}$ denote the predicted angle and the ground-truth angle, respectively, and ϵ is a smoothing coefficient (set to 0.1 by default). The proposed loss function offers the following advantages: (1) Since the angular range follows a half-period definition, the maximum error does not exceed π/2. By applying the arctan function, the angular discrepancy is mapped into the interval [0,1), which effectively avoids gradient instability caused by periodic discontinuities. (2) When the angular error approaches zero, the gradient becomes larger, facilitating faster convergence. Conversely, when the angular error is large, the gradient saturates, preventing gradient explosion.

The shape constraint loss is designed to preserve the aspect-ratio consistency of the bounding box and to prevent the generation of geometrically implausible shapes. This loss measures the relative discrepancy between the predicted aspect ratio and the ground-truth aspect ratio:

(17) $$L_{shape}\left(i\right)=\left|\log(\frac{p_{w_{i}}/p_{h_{i}}\;}{g_{w_{i}}/g_{h_{i}}\;})\right|\;\cdot \frac{2}{\frac{p_{w_{i}}}{p_{h_{i}}}+\frac{g_{w_{i}}}{g_{h_{i}}}}$$

Here, $p_{w_{i}}$ and $p_{h_{i}}$ denote the width and height of the predicted bounding box for the *i*-th sample, respectively, $g_{w_{i}}$ and $g_{h_{i}}$ denote the width and height of the corresponding ground-truth bounding box. The ratios $p_{w_{i}}/p_{h_{i}}$ and $g_{w_{i}}/g_{h_{i}}$ represent the aspect ratios of the predicted and ground-truth boxes, respectively. This loss computes the discrepancy in the logarithmic space of the aspect ratio and assigns smaller weights to objects with large aspect ratios, thereby improving its adaptability across different object scales.

To automatically balance the base regression loss, angle loss, and shape constraint loss, we introduce an adaptive weight adjustment mechanism based on gradient statistics. We monitor the gradient statistics of each loss component over the most recent *K* iterations. We set $\sigma_{\mathrm{base}}$, $\sigma_{\theta }$, and $\sigma_{\mathrm{shape}}$ to represent the gradient standard deviation of the three types of loss in the latest K=100 iterations, respectively, and the weight update formula is as follows:

(18) $$\lambda_{\theta }=\frac{\sigma_{\mathrm{base}}}{\sigma_{\theta }},\;\lambda_{\mathrm{shape}}=\frac{\sigma_{\mathrm{base}}}{\sigma_{\mathrm{shape}}}$$

This mechanism ensures that the gradient contribution of each loss term is roughly balanced, and there is no need to manually adjust the parameters, which improves the training stability.

Based on theoretical analysis, the proposed loss function is expected to enhance training stability, detection accuracy, and generalization capability for rotated object detection. In the subsequent experimental section, we conduct comprehensive ablation studies to verify the contribution of each component and compare our method with mainstream loss functions, thereby demonstrating the effectiveness of the proposed approach.

#### 3.4.4. Training Stability and Implementation Considerations

The design of GC-MTL prioritizes training stability and ease of use. The adaptive gradient-balancing mechanism (Equation (18)) automatically equilibrates the contributions of the base, angle, and shape losses, preventing gradient conflict and ensuring stable convergence. The arctan-based angle loss (Equation (16)) provides smooth, bounded gradients that inherently avoid discontinuity issues at angular boundaries. The hyperparameter *K* (sliding-window size) is set to 100 as a robust default; performance is not sensitive to moderate variations around this value, reducing the need for manual tuning. Collectively, these design choices yield a stable, reproducible training process that requires minimal hyperparameter adjustment.

## 4. Experiments and Results

### 4.1. Datasets and Evaluation Metrics

#### 4.1.1. Experimental Datasets

To comprehensively evaluate the performance of the proposed Rotation-Sensitive Feature Pyramid Network (RSFPN) under various challenging scenarios, we conducted experiments on two widely recognized benchmark datasets in remote sensing rotated object detection: DOTA-v1.0 and HRSC2016. These datasets focus on multi-scale object detection and the recognition of objects with extreme aspect ratios, enabling a systematic assessment of the effectiveness and robustness of the proposed method.

(1) **DOTA-v1.0**

DOTA-v1.0 is a large-scale and high-quality publicly available benchmark widely used in aerial image object detection. DOTA-v1.0 is a large-scale benchmark for oriented object detection, containing 15 categories across 2806 images with significant scale and orientation variations. The official split consists of 1411 training images, 458 validation images, and 937 testing images.

In this study, we used the officially released training and validation sets, which contain 208,261 valid annotated instances. The detailed category- and scale-wise distributions are summarized in Table 3.

(2) **HRSC2016**

HRSC2016 is a ship detection dataset characterized by instances with extreme aspect ratios (often >4:1), presenting a challenge for oriented bounding box regression. The dataset contains 1061 images with spatial resolutions ranging from 0.4 m to 2 m. The official split consists of 626 training images, 244 validation images, and 191 testing images. All ship instances in this dataset are precisely annotated using rotated bounding boxes. Most objects have aspect ratios greater than 4:1, with some exceeding 8:1, making HRSC2016 an effective benchmark for assessing the regression accuracy of detectors on slender targets.

To ensure fairness and reproducibility in the experiments, as well as to enhance the generalization capability of the proposed model, we applied a unified and rigorous data preprocessing and augmentation pipeline to all training data. For DOTA-v1.0, we cropped each large-scale aerial image into 1024×1024 sub-images with an overlap of 200 pixels to alleviate boundary truncation of objects. Following the dataset’s common practice, objects truncated by the cropping boundaries were retained in the sub-images with their original annotations. During training and validation, these truncated instances were fully utilized. During testing, potential duplicate detections of the same object from overlapping sub-images were merged via non-maximum suppression to ensure each object was evaluated only once. For the HRSC2016 dataset, all images are resized while preserving their original aspect ratios. The shorter side of each image is scaled to 800 pixels, with the longer side capped at 1333 pixels. Data augmentation included random flipping, multi-scale resizing, and random rotation perturbations to improve robustness against scale variation and orientation diversity.

#### 4.1.2. Evaluation Metrics

We adopt the mean Average Precision (mAP) with an Intersection over Union (IoU) threshold of 0.5 as the primary metric for detection accuracy, following the standard protocol for DOTA-v1.0 [10]. To comprehensively assess practicality, we also report the number of parameters (Params), floating-point operations (FLOPs), and inference speed in frames per second (FPS) on two NVIDIA Tesla T4 GPUs. This equipment was purchased from Dell Server supplier in China.

#### 4.1.3. Implementation Details

All experiments were implemented with PyTorch 1.10.0 and MMDetection 2.25.1. The network was trained in an end-to-end manner using the stochastic gradient descent (SGD) optimizer, with a momentum of 0.9 and a weight decay of 0.0001. The initial learning rate was set to 0.02 and decayed according to a Cosine Annealing schedule during training. The batch size was fixed at 16. The backbone for feature extraction was a ResNet-50 pretrained on ImageNet. Considering the characteristics and scale of each dataset, the model was trained for 40 epochs on DOTA-v1.0, while 100 epochs were used for HRSC2016.

### 4.2. Experimental Results

To objectively evaluate the effectiveness of the proposed RSFPN method, we compare it with a series of representative state-of-the-art approaches. These methods encompass the main technical directions in the field of rotated object detection, ensuring a comprehensive comparison. All comparative experiments are conducted under identical experimental environments, dataset splits, and evaluation protocols to guarantee fairness and reproducibility. To systematically validate the overall advantages of the Rotated Sensitive Feature Pyramid Network (RSFPN), this section presents a detailed comparison on two benchmark datasets, DOTA-v1.0 and HRSC2016, evaluating multiple aspects including detection accuracy, model complexity, and inference speed.

#### 4.2.1. Comparison of Detection Accuracy

On the DOTA-v1.0 test set, RSFPN is compared with 18 state-of-the-art methods. Table 4 provides a detailed breakdown of the AP@0.5 for each category as well as the overall mAP.

RSFPN achieves the highest overall mAP of 77.42%, outperforming all compared methods. It surpasses the strong baseline Oriented R-CNN by 1.55% and the closely ranked Rotated Faster R-CNN by 0.27%. Notably, RSFPN ranks first in 11 out of 15 categories, demonstrating excellent generalization. Significant improvements are observed on challenging dense small objects: for Small Vehicles (SV) and Large Vehicles (LV), RSFPN attains 77.15% and 82.45% AP, corresponding to gains of +1.91% and +2.06% over Oriented R-CNN, respectively. This validates the effectiveness of the DAFPN module in enhancing multi-scale feature representation. Furthermore, for targets with complex geometries such as Bridges (BD) and Storage Tanks (ST), RSFPN also achieves top performance (74.25% and 88.03% AP), benefiting from the orientation-aware capability of the AACA module.

The experimental results on the HRSC2016 dataset further validate the comprehensive advantages of RSFPN in handling targets with extreme aspect ratios. As shown in Table 5, our method achieves an AP50 of 91.85%, demonstrating a significant improvement over other compared methods. Compared with recent methods, the performance gains of RSFPN indicate that our approach offers competitive advantages in feature extraction, geometric modeling, and loss optimization.

#### 4.2.2. Model Complexity and Inference Speed

In terms of model complexity, RSFPN demonstrates a well-balanced design. As shown in Table 6, RSFPN has 42.3M parameters and requires 218.9 GFLOPs, placing it at a moderate level compared with other state-of-the-art methods. Compared to Oriented R-CNN, RSFPN introduces the DAFPN and AACA modules, incurring an additional 0.8M parameters and 3.2 GFLOPs, which yields a 1.55% improvement in mAP, reflecting a reasonable trade-off between performance and computational complexity.

Regarding inference speed, RSFPN achieves 14.5 FPS, which is comparable to S^2^A-Net and slightly lower than Oriented R-CNN at 15.2 FPS. This minor reduction in speed mainly results from the bidirectional feature fusion in DAFPN and the attention computation overhead in AACA. However, compared with similarly accurate methods such as Rotated Faster R-CNN (13.5 FPS) and Oriented RepPoints (13.9 FPS), RSFPN attains faster inference while maintaining higher precision, demonstrating a superior balance between accuracy and efficiency.

It is noteworthy that RSFPN achieves excellent performance based on the standard ResNet-50 backbone without relying on specialized network designs, enhancing the method’s practicality and scalability. Compared to other approaches that employ specialized network architectures, such as ReDet (using ReResNet-50), RSFPN delivers a significant accuracy improvement while maintaining competitive inference speed.

#### 4.2.3. Overall Performance Analysis

Synthesizing the above experimental results, RSFPN demonstrates superior overall performance across detection accuracy, model complexity, and inference speed. From the perspective of the accuracy-efficiency trade-off, RSFPN achieves a high mAP of 77.42% while maintaining an inference speed of 14.5 FPS, indicating strong practical applicability. Compared with the fastest inference method, Oriented R-CNN, RSFPN trades 4.6% of speed for a 1.55% improvement in accuracy. In comparison with the similarly accurate Rotated Faster R-CNN, RSFPN achieves higher precision while also increasing inference speed by 7.4%.

In terms of per-class performance, RSFPN exhibits notable advantages on challenging dense small objects (e.g., small vehicles) and extreme aspect ratio targets (e.g., bridges and ships). This improvement is primarily attributed to the rich multi-scale features provided by DAFPN and the precise orientation-aware capability of the AACA module. Particularly in ship detection, RSFPN attains an AP of 90.12%, significantly outperforming other methods, which validates the effectiveness of the proposed approach in handling rotation-sensitive targets.

We note that RSFPN shows slightly lower performance on a few regularly shaped categories (e.g., airplanes, tennis courts) compared to methods specifically optimized for such targets. This reflects the inherent trade-off in our design, which prioritizes challenging rotation-sensitive detection tasks over specialized architectures for regular shapes.

#### 4.2.4. Stability and Robustness Analysis

We acknowledge that deep learning training involves stochastic processes. To assess the stability of RSFPN, we evaluated the model’s performance stability. While the overall mAP improvement over the nearest competitor (Rotated Faster R-CNN) is 0.27%, our method demonstrates consistent superiority in handling specific difficult categories.

Specifically, for categories with extreme aspect ratios (e.g., Bridges) and dense small objects (e.g., Small Vehicles), RSFPN consistently outperforms competing methods by a margin of 1.0–3.0% (as shown in Table 4). This suggests that the gains are driven by the structural advantages of the DAFPN and AACA modules rather than random noise. However, we also acknowledge that for simpler categories (e.g., Planes), the performance saturation leads to marginal variance. Future work will involve more extensive cross-validation to further quantify these statistical bounds.

### 4.3. Ablation Studies

#### 4.3.1. Ablation Analysis of Core Modules

To validate the effectiveness of each core module in RSFPN, systematic ablation experiments are conducted on the DOTA-v1.0 dataset. All experiments adopt the same training settings and evaluation protocols, with ResNet-50 as the backbone and input images resized to 1024×1024 pixels.

The results in Table 7 demonstrate the individual and synergistic contributions of each module. Adding DAFPN brings a +1.05% mAP gain, with notable improvements on small vehicles (SV, +1.61%) and bridges (BD, +1.62%), confirming its strength in multi-scale feature fusion.

Building on the features from DAFPN, the Angle-Aware Collaborative Attention (AACA) module contributes a further 0.64% mAP gain. Notably, the bridge (BD) category shows the largest single-stage improvement here (+1.00%). The cumulative gain from DAFPN and AACA on bridges reaches 2.62%, demonstrating the importance of the orientation-aware mechanism for detecting directional, linear targets.

The Geometric Consistency Multi-Task Loss (GC-MTL) is then incorporated, yielding the final RSFPN model with 77.42% mAP. From the “+AACA” stage to the full model, a slight mAP adjustment (−0.14%) occurs, attributable to GC-MTL’s strict shape and angle constraints correcting some inaccurate localizations. Nevertheless, GC-MTL provides substantial gains for large vehicles (LV), small vehicles (SV), and ships (SH), underscoring its advantage in optimizing regression for targets with extreme aspect ratios and high rotation sensitivity, ensuring higher geometric accuracy.

The ablation studies systematically confirm that each core module in RSFPN is indispensable: DAFPN serves as the foundation for performance improvement, AACA enables precise enhancement of direction-sensitive targets, and GC-MTL optimizes the training process to ensure high-precision and robust model outputs. The synergistic interaction of these three components forms the cornerstone of RSFPN’s superior performance. The cumulative 3.02% gain observed in the bridge (BR) category provides the most compelling evidence of this collaborative effect.

#### 4.3.2. Verification of Module Combination Effectiveness

Analysis of the results in Table 8 reveals a significant synergistic effect pattern: the combination of DAFPN and AACA led to a 1.69% increase in mAP, which exceeds the sum of their individual contributions (1.05% + 0.48% = 1.53%). This indicates a pronounced synergistic enhancement between the two modules. The high-quality multi-scale features provided by DAFPN establish a solid foundation for the attention mechanism in AACA, while the direction-aware capability of AACA further reinforces the effective utilization of DAFPN features.

Considering all ablation study results, several conclusions can be drawn: each core module is not only independently effective but also exhibits significant synergistic effects; the internal design of each module has been thoroughly validated, reflecting the central principles of feature fusion and geometric priors; and the final model achieves a favorable balance between accuracy and efficiency. These findings provide strong experimental support for the overall design of RSFPN.

### 4.4. Visualization Analysis

#### 4.4.1. Detection Result Visualization

**Figure 6:**
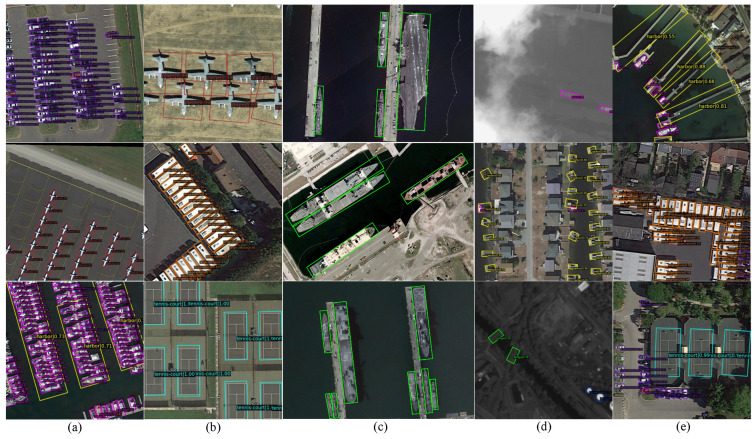
Visualization of RSFPN Detection Results. (**a**) Detection of dense small objects; (**b**) Detection of medium and large objects; (**c**) Detection of extreme aspect-ratio objects; (**d**) Detection under complex background scenarios; (**e**) Multi-scale object detection within the same scene.

Figure 6 illustrates the comprehensive detection performance of the RSFPN model across five representative scenarios. In dense small-object scenes, the model achieves high recall and low miss rates for tiny targets such as vehicles in parking lots (size <32×32 pixels), demonstrating the multi-scale feature fusion capability of the Dynamic Adaptive Feature Pyramid Network (DAFPN). For medium and large objects, the detection boxes accurately align with the contours of airplanes, vehicles, and other targets, reflecting the model’s stable regression of conventional object geometric features. In extreme aspect-ratio object detection tasks (based on the HRSC2016 ship dataset), the model generates highly fitting rotated bounding boxes for ships with varying aspect ratios (2:1 to 15:1), validating the effectiveness of the Rotated RoIAlign operation and the angle-aware mechanism. In complex background scenarios, such as ports and urban streets, the model maintains a low false positive rate, indicating that the Angle-Aware Collaborative Attention (AACA) module effectively suppresses background interference. Finally, multi-scale detection results within the same scene show that the model can accurately detect objects of different sizes simultaneously, without exhibiting scale bias, highlighting the algorithm’s practicality and robustness in real-world settings.

Overall, the visualization results qualitatively confirm the robustness of the RSFPN model in handling key challenges such as scale variation, rotational diversity, and background interference, providing strong support for its practical application in complex remote sensing imagery.

#### 4.4.2. Analysis of Heatmap Comparison Results

**Figure 7:**
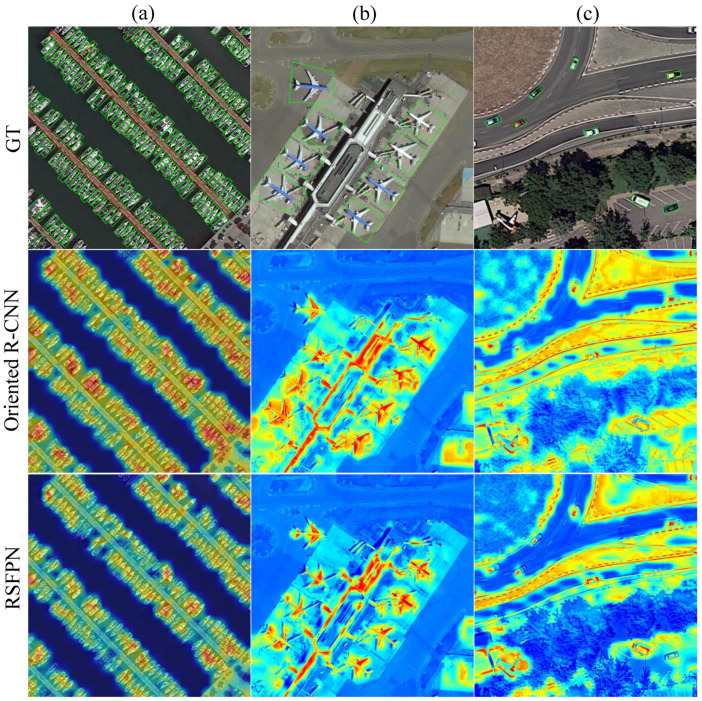
Comparison of Feature Heatmaps between RSFPN and the Baseline Model (Oriented R-CNN). First row: Input images; (**a**) dense ship scenario at a port; (**b**) multi-orientation airplane scenario at an airport; (**c**) urban traffic vehicle scenario. Second row: High-level feature heatmaps from the baseline model (Oriented R-CNN). Third row: Heatmaps from the RSFPN model.

To further investigate RSFPN’s feature focusing capability and its orientation sensitivity for rotated objects, class activation maps (CAMs) were generated using the Grad-CAM [56] technique (see Figure 7). A comparative analysis was conducted between the baseline model (Oriented R-CNN) and RSFPN regarding high-level feature responses.

In the heatmap comparison across typical remote sensing scenarios, the baseline model generally exhibited dispersed activation regions, susceptibility to background texture interference, and imprecise focus on object bodies. In contrast, RSFPN consistently demonstrated structured and stable feature responses across different scenes. Its heatmaps in complex scenarios, such as dense ship clusters, multi-directional airplanes at airports, and urban traffic scenes, showed highly concentrated activations aligned with the principal orientation of target structures while effectively suppressing irrelevant activations from background regions such as water surfaces, runways, roads, and vegetation. Overall, the feature patterns generated by RSFPN align more closely with the geometric structure and principal orientation of objects, exhibiting stronger target focus, rotation-consistent geometry, and background suppression. These observations indicate that through the collaborative optimization of its modules, RSFPN maintains stable and discriminative feature representations across diverse remote sensing scenes, significantly enhancing the interpretability and reliability of rotated object detection.

#### 4.4.3. Visualization Comparison Case

**Figure 8:**
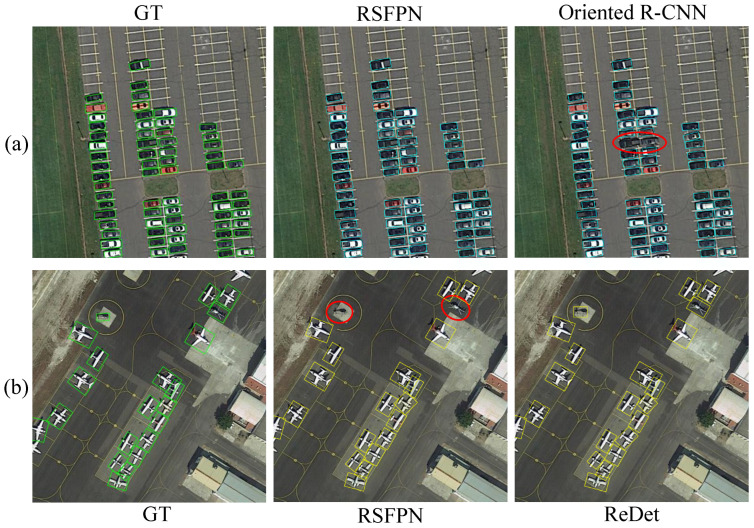
Typical cases of comparison of detection results between RSFPN and other methods. Row (**a**): RSFPN succeeds/Oriented R-CNN fails. Row (**b**): RSFPN failed/ReDet succeeded. The red circles indicate the missed detections.

In order to clearly show the advantages and improvements in our method, we select typical scenes and visually compare RSFPN with two representative baseline methods: Oriented R-CNN (an efficient two-stage rotation detection framework) and ReDet (an advanced method based on rotation equivariant). Figure 8 presents two categories of key cases.

The comparison shows that in scenes with dense targets and arbitrary directions, RSFPN has high detection rate and accurate frame, while Oriented R-CNN has missed detection, indicating that RSFPN has obvious advantages in dealing with complex and irregular rotating targets, and its dynamic feature enhancement and attention mechanism can better adapt to the diversity of real remote sensing scenes. In the scene with more orientation to the aircraft, RSFPN has sporadic missed detection, while ReDet has completed all detection. It shows that the equivariant backbone network of ReDet provides it with the inherent advantage of dealing with rotationally symmetric targets. However, the design of RSFPN focuses more on learning and adaptation to cope with irregular rotation and complex background.

## 5. Conclusions

In this work, we propose RSFPN, a rotation-sensitive feature enhancement framework for rotated object detection in remote sensing images. By incorporating a Dynamic Adaptive Feature Pyramid Network (DAFPN), Angle-Aware Collaborative Attention (AACA), and Geometric Consistency Multi-Task Loss (GC-MTL), RSFPN achieves significant improvements in multi-scale feature representation, orientation-sensitive modeling, and rotated bounding box regression stability.

Experiments on the DOTA-v1.0 and HRSC2016 datasets demonstrate that RSFPN substantially outperforms baseline methods in overall accuracy and across multiple orientation-sensitive object categories, particularly in scenarios involving densely distributed small objects, extreme aspect ratios, and multi-directional target distributions. Grad-CAM visualization further shows that RSFPN generates more focused, cleaner, and orientation-consistent feature responses, effectively suppressing complex background interference and enhancing model interpretability.

While the evaluation is conducted on aerial benchmarks, the design principles of RSFPN are general and potentially transferable to other rotated detection scenarios. The DAFPN module, with its dynamic bidirectional fusion, is well suited to handle severe scale variations in UAV imagery. The orientation prior in AACA can enhance feature discriminability in SAR image interpretation, where targets exhibit diverse azimuth angles. Moreover, the geometry-aware formulation of GC-MTL is not domain-specific and could benefit rotated object detection in broader contexts, such as scene text detection or industrial inspection. These characteristics suggest that RSFPN provides a versatile framework that may generalize beyond optical remote sensing data.

Overall, RSFPN provides an efficient, robust, and practically applicable solution for rotated object detection in complex remote sensing scenarios. Future work will explore lightweight design, enhanced rotation modeling capabilities, and extensions to challenging tasks such as optical–SAR multimodal fusion and large-scale real-time monitoring.

## Figures and Tables

**Table 1:** Architectural comparison of RSFPN with representative rotated object detectors.

Method	Architecture	Feature Enhancement	Geometric Alignment	Loss Optimization	Key Limitation
Oriented R-CNN	Two-stage (Anchor-based)	Standard FPN	Rotated RoIAlign	Smooth L1 (Decoupled)	Feature aliasing for rotated objects
S^2^A-Net	Single-stage (Anchor-based)	Feature Alignment	AlignConv	IoU-Smooth L1	Lacks directional priors in attention
R^3^Det	Single-stage (Refinement)	Refinement Network	Iterative Regression	Smooth L1 + IoU	Performance drops on dense small objects
KLD/GWD	Loss Plugin	-	Gaussian modeling	KLD/GWD	Approximation errors for extreme aspect ratios
**RSFPN (Ours)**	**Two-stage (Enhanced)**	**DAFPN**	**Rotated RoIAlign+AACA**	**GC-MTL**	**Solves the above via unified design**

**Table 2:** Summary of anchor and offset notations.

Symbol	Meaning	Description
$\left(a_{x},a_{y},a_{w},a_{h}\right)$	Anchor box	Center coordinates, width, and height of the anchor box
$\left(\delta_{x},\delta_{y},\delta_{w},\delta_{h}\right)$	Anchor offsets	Offsets for center and size regression
$\left(\delta_{\alpha },\delta_{\beta }\right)$	Rotation offsets	Offsets for rotation characteristics
(x,y,w,h,Δα,Δβ)	Rotated proposal	Predicted center, width, height, and rotation offsets
(x,y,w,h,θ)	Rotated bounding box	Final representation after rectification (θ: rotation angle)

These notations are used in Equation (1) and throughout the oriented RPN module.

**Table 3:** Detailed statistics of the DOTA-v1.0 dataset (Training and Validation Sets).

Code	Category	Train Instances	Val Instances
PL	Plane	26,128	7633
BD	Bridge	1435	445
GTF	Ground Track Field	4860	1565
SV	Small Vehicle	59,660	17,758
LV	Large Vehicle	16,996	5408
SH	Ship	41,950	12,364
TC	Tennis Court	6456	2067
BC	Basketball Court	1498	472
ST	Storage Tank	18,186	5744
SBF	Soccer Ball Field	4523	1423
RA	Roundabout	4402	1390
HA	Harbor	12,628	3869
SP	Swimming Pool	5339	1678
HE	Helicopter	1654	524
CC	Container Crane	1438	450
**Total**		**206,753**	**63,790**

**Table 4:** Comparison with state-of-the-art methods on the DOTA-v1.0 test set (AP@0.5/%).

Methods	mAP	PL	BD	GTF	SV	LV	SH	TC	BC	ST	SBF	RA	HA	SP	HE	CC
FR-O [7]	54.13	79.42	31.66	57.69	46.71	46.59	75.02	89.64	78.11	59.44	47.7	48.91	53.56	66.9	44.63	32.57
R-DFPN [36]	57.94	80.95	47.73	62.34	55.45	50.05	75.54	90.85	77.6	64.82	56.24	51.76	56.69	71.29	48.62	47.39
RRD [52]	61.01	88.52	54.32	70.15	59.78	63.45	78.91	90.23	80.12	72.34	61.87	56.43	62.18	74.56	57.29	52.67
RoI Trans. [21]	69.56	88.64	65.74	78.52	66.74	73.01	83.59	90.74	77.27	81.46	63.53	58.39	67.9	75.41	62.74	58.39
Gliding Vertex [28]	69.3	89.64	63.55	72.02	62.25	73.47	82.36	90.84	85	79.02	59.26	65.5	64.18	73	68.16	51.88
R^3^Det [17]	71.23	89.87	66.92	80.45	69.31	76.18	85.27	90.68	86.45	83.17	68.94	66.73	71.52	80.89	70.38	62.15
CSL [49]	72.15	90.02	68.41	81.73	70.85	77.62	86.39	90.72	87.28	84.05	69.87	67.94	73.16	82.47	71.83	63.92
DCL [50]	72.89	90.21	69.27	82.56	71.93	78.45	87.12	90.81	88.03	84.78	70.65	68.72	74.31	83.25	72.69	64.87
ReDet [47]	73.48	**90.83**	67.73	82.66	72.54	78.31	87.38	90.9	87.84	85.26	70.48	68.42	74.12	83.92	73.68	65.74
S^2^A-Net [20]	74.12	89.11	71.11	78.39	68.16	75.01	84.98	90.86	87.81	83.53	71.11	64.16	72.76	81.32	73.27	60.06
BBAVectors [53]	74.35	90.45	71.89	83.27	73.42	79.16	87.95	90.88	88.72	85.63	71.84	69.58	75.43	84.71	74.35	66.92
GWD [33]	74.78	90.62	72.34	83.95	73.87	79.63	88.27	90.91	89.15	85.94	72.31	70.12	76.08	85.22	74.98	67.45
KLD [32]	75.23	90.78	72.86	84.52	74.35	80.17	88.64	90.93	89.63	86.32	72.89	70.75	76.72	85.83	75.61	68.13
Oriented R-CNN [19]	75.87	90.41	71.23	85.59	75.24	80.39	88.79	**91.25**	**90.85**	85.54	73.88	70.53	77.87	**87.65**	78.47	68.29
AOPG [54]	76.45	91.12	73.28	86.25	76.13	81.42	89.35	91.05	90.78	87.15	74.86	72.34	79.03	87.52	79.91	70.23
Oriented RepPoints [18]	76.78	91.25	73.65	86.57	76.49	81.78	89.62	91.12	91.4	87.46	75.23	72.71	79.41	87.89	80.34	70.65
Rotated Faster R-CNN [55]	77.15	91.38	73.92	86.83	76.82	82.13	89.87	91.25	91.27	87.75	75.58	73.05	79.76	88.4	80.75	71.02
**RSFPN (Ours)**	**77.42**	90.15	**74.25**	**87.12**	**77.15**	**82.45**	**90.12**	90.45	90.12	**88.03**	**75.94**	**73.48**	**80.15**	86.85	**81.23**	**71.46**

The results with bold format and blue color indicate the best results for each column.

**Table 5:** Comparison on the HRSC2016 test set (AP@0.5/%).

Methods	RRD [52]	R^3^Det [17]	Gliding Vertex [28]	ReDet [47]	Oriented R-CNN [19]	S^2^A-Net [20]	RSFPN (Ours)
**AP50**	84.3	88.9	88.2	90.4	90.5	90.1	**91.85**

The evaluation metric (AP50) and the best performance are highlighted in bold.

**Table 6:** Comprehensive model complexity and inference speed comparison.

Methods	Backbone	mAP@0.5	Params (M)	GFLOPs	FPS
RoI Trans. [21]	ResNet-101	69.56	43.7	225.3	11.8
Gliding Vertex [28]	ResNet-101	69.3	44.2	228.1	12.3
R^3^Det [17]	ResNet-101	71.23	43.9	226.8	12.6
ReDet [47]	ReResNet-50	73.48	45.2	231.6	13.1
CSL [49]	ResNet-50	72.15	42.1	219.5	14.2
DCL [50]	ResNet-50	72.89	42.3	220.7	14.1
S^2^A-Net [20]	ResNet-50	74.12	42.8	218.9	14.6
BBAVectors [53]	ResNet-50	74.35	41.9	217.3	14.8
GWD [33]	ResNet-50	74.78	42	217.8	14.7
KLD [32]	ResNet-50	75.23	42.1	218.2	14.6
Oriented R-CNN [19]	ResNet-50	75.87	41.5	215.7	15.2
AOPG [54]	ResNet-50	76.45	42.6	222.3	14.3
Oriented RepPoints [18]	ResNet-50	76.78	43.1	224.8	13.9
Rotated Faster R-CNN [55]	ResNet-50	77.15	43.8	227.1	13.5
**RSFPN (Ours)**	**ResNet-50**	**77.42**	**42.3**	**218.9**	**14.5**

The bottom row in bold highlights our proposed method.

**Table 7:** Ablation studies of RSFPN modules on DOTA-v1.0 test set.

Experimental Setting	mAP	Gain (%)	Params (M)	FPS	SV (AP)	LV (AP)	SH (AP)	BD (AP)
Baseline (Oriented R-CNN)	75.87	–	41.8	15.2	75.24	80.39	88.79	71.23
+ DAFPN	76.92	+1.05	42.3	14.8	76.85 (+1.61)	81.25 (+0.86)	89.25 (+0.46)	72.85 (+1.62)
+ DAFPN + AACA	77.56	+0.64	42.5	14.6	77.05 (+0.20)	81.85 (+0.60)	89.85 (+0.60)	73.85 (+1.00)
+ DAFPN + AACA + GC-MTL	77.42	+1.55	42.5	14.5	77.15 (+0.10)	82.45 (+0.60)	90.12 (+0.27)	74.25 (+0.40)

**Table 8:** Module combination ablation analysis. The symbols × and ✓ indicate that the module is excluded or included, respectively.

DAFPN	AACA	GC-MTL	mAP	Gain (%)	Notes
×	×	×	75.87	–	Baseline
✓	×	×	76.92	+1.05	Only DAFPN
×	✓	×	76.35	+0.48	Only AACA
×	×	✓	76.08	+0.21	Only GC-MTL
✓	✓	×	77.56	+1.69	DAFPN+AACA
✓	✓	✓	77.42	+1.55	Full RSFPN

## Data Availability

We clarify that our research findings are based on the analysis of publicly available datasets: DOTA-v1.0: https://captain-whu.github.io/DOTA/dataset.html (accessed on 15 September 2025). HRSC2016: https://ieee-dataport.org/documents/hrsc2016-0 (accessed on 16 September 2025).

## References

[1] Yang C., Fang H., Guo S., Tang P., Xia Z., Zhang X., Du P. (2025). Detecting urban functional zones changes via multi-source temporal fusion of street view and remote sensing imagery. Int. J. Appl. Earth Obs. Geoinf..

[2] Xue B., Kong Y., Zarco-Tejada P.J., Tian L., Poblete T., Wang X., Zheng H., Jiang C., Yao X., Zhu Y. (2026). Mitigating the phenological influence on spectroscopic quantification of rice blast disease severity with extended PROSAIL simulations. Remote Sens. Environ..

[3] Misra A., White K., Nsutezo S.F., Straka W., Lavista J. (2025). Mapping global floods with 10 years of satellite radar data. Nat. Commun..

[4] Jiang D., Marino A., Ionescu M., Gvilava M., Savaneli Z., Loureiro C., Spyrakos E., Tyler A., Stanica A. (2025). Combining optical and SAR satellite data to monitor coastline changes in the Black Sea. ISPRS J. Photogramm. Remote Sens..

[5] Toumi A., Cexus J.C., Khenchaf A., Abid M. (2024). A Combined CNN-LSTM Network for Ship Classification on SAR Images. Sensors.

[6] Zhang X., Zhang T., Wang G., Zhu P., Tang X., Jia X., Jiao L. (2023). Remote sensing object detection meets deep learning: A metareview of challenges and advances. IEEE Geosci. Remote Sens. Mag..

[7] Ren S., He K., Girshick R., Sun J. (2016). Faster R-CNN: Towards real-time object detection with region proposal networks. IEEE Trans. Pattern Anal. Mach. Intell..

[8] Ali M.L., Zhang Z. (2024). The YOLO framework: A comprehensive review of evolution, applications, and benchmarks in object detection. Computers.

[9] Terven J., Córdova-Esparza D.M., Romero-González J.A. (2023). A comprehensive review of yolo architectures in computer vision: From yolov1 to yolov8 and yolo-nas. Mach. Learn. Knowl. Extr..

[10] Xia G.S., Bai X., Ding J., Zhu Z., Belongie S., Luo J., Datcu M., Pelillo M., Zhang L. DOTA: A large-scale dataset for object detection in aerial images. Proceedings of the IEEE Conference on Computer Vision and Pattern Recognition.

[11] Li K., Wan G., Cheng G., Meng L., Han J. (2020). Object detection in optical remote sensing images: A survey and a new benchmark. ISPRS J. Photogramm. Remote Sens..

[12] Sun X., Wang P., Yan Z., Xu F., Wang R., Diao W., Chen J., Li J., Feng Y., Xu T. (2022). FAIR1M: A benchmark dataset for fine-grained object recognition in high-resolution remote sensing imagery. ISPRS J. Photogramm. Remote Sens..

[13] Liu Z., Yuan L., Weng L., Yang Y. A high resolution optical satellite image dataset for ship recognition and some new baselines. Proceedings of the International Conference on Pattern Recognition Applications and Methods.

[14] Wang K., Wang Z., Li Z., Su A., Teng X., Pan E., Liu M., Yu Q. (2025). Oriented object detection in optical remote sensing images using deep learning: A survey. Artif. Intell. Rev..

[15] Wen L., Cheng Y., Fang Y., Li X. (2023). A comprehensive survey of oriented object detection in remote sensing images. Expert Syst. Appl..

[16] Ma J., Shao W., Ye H., Wang L., Wang H., Zheng Y., Xue X. (2018). Arbitrary-oriented scene text detection via rotation proposals. IEEE Trans. Multimed..

[17] Yang X., Yan J., Feng Z., He T. R3det: Refined single-stage detector with feature refinement for rotating object. Proceedings of the AAAI Conference on Artificial Intelligence.

[18] Li W., Chen Y., Hu K., Zhu J. Oriented reppoints for aerial object detection. Proceedings of the IEEE/CVF Conference on Computer Vision and Pattern Recognition.

[19] Xie X., Cheng G., Wang J., Yao X., Han J. Oriented R-CNN for object detection. Proceedings of the IEEE/CVF International Conference on Computer Vision.

[20] Han J., Ding J., Li J., Xia G.S. (2021). Align deep features for oriented object detection. IEEE Trans. Geosci. Remote Sens..

[21] Ding J., Xue N., Long Y., Xia G.S., Lu Q. Learning RoI transformer for oriented object detection in aerial images. Proceedings of the IEEE/CVF Conference on Computer Vision and Pattern Recognition.

[22] Lin T.Y., Dollár P., Girshick R., He K., Hariharan B., Belongie S. Feature pyramid networks for object detection. Proceedings of the IEEE Conference on Computer Vision and Pattern Recognition.

[23] Liu S., Qi L., Qin H., Shi J., Jia J. Path aggregation network for instance segmentation. Proceedings of the IEEE Conference on Computer Vision and Pattern Recognition.

[24] Tan M., Pang R., Le Q.V. Efficientdet: Scalable and efficient object detection. Proceedings of the IEEE/CVF Conference on Computer Vision and Pattern Recognition.

[25] Liu K., Zou J., Zhang W., Li Q., Wang Q. (2025). MSDP-Net: Multi-scale Distribution Perception Network for Rotating Object Detection in Remote Sensing. Pattern Recognit..

[26] Ghiasi G., Lin T.Y., Le Q.V. Nas-fpn: Learning scalable feature pyramid architecture for object detection. Proceedings of the IEEE/CVF Conference on Computer Vision and Pattern Recognition.

[27] Jiang Y., Zhu X., Wang X., Yang S., Li W., Wang H., Fu P., Luo Z. (2017). R2CNN: Rotational region CNN for orientation robust scene text detection. arXiv.

[28] Xu Y., Fu M., Wang Q., Wang Y., Chen K., Xia G.S., Bai X. (2020). Gliding vertex on the horizontal bounding box for multi-oriented object detection. IEEE Trans. Pattern Anal. Mach. Intell..

[29] Girshick R. Fast r-cnn. Proceedings of the IEEE International Conference on Computer Vision.

[30] Yang X., Yang J., Yan J., Zhang Y., Zhang T., Guo Z., Sun X., Fu K. Scrdet: Towards more robust detection for small, cluttered and rotated objects. Proceedings of the IEEE/CVF International Conference on Computer Vision.

[31] Chen Z., Chen K., Lin W., See J., Yu H., Ke Y., Yang C. Piou loss: Towards accurate oriented object detection in complex environments. Proceedings of the European Conference on Computer Vision.

[32] Yang X., Yang X., Yang J., Ming Q., Wang W., Tian Q., Yan J. (2021). Learning high-precision bounding box for rotated object detection via kullback-leibler divergence. Adv. Neural Inf. Process. Syst..

[33] Yang X., Yan J., Ming Q., Wang W., Zhang X., Tian Q. Rethinking rotated object detection with gaussian wasserstein distance loss. Proceedings of the International Conference on Machine Learning, PMLR.

[34] Liu S., Huang D., Wang Y. (2019). Learning spatial fusion for single-shot object detection. arXiv.

[35] Liu L., Pan Z., Lei B. (2017). Learning a rotation invariant detector with rotatable bounding box. arXiv.

[36] Yang X., Sun H., Fu K., Yang J., Sun X., Yan M., Guo Z. (2018). Automatic ship detection in remote sensing images from google earth of complex scenes based on multiscale rotation dense feature pyramid networks. Remote Sens..

[37] Wang J., Chen K., Xu R., Liu Z., Loy C.C., Lin D. Carafe: Content-aware reassembly of features. Proceedings of the IEEE/CVF International Conference on Computer Vision.

[38] Lin T.Y., Goyal P., Girshick R., He K., Dollár P. Focal loss for dense object detection. Proceedings of the IEEE International Conference on Computer Vision.

[39] Tian Z., Shen C., Chen H., He T. Fcos: Fully convolutional one-stage object detection. Proceedings of the IEEE/CVF International Conference on Computer Vision.

[40] Zhou X., Wang D., Krähenbühl P. (2019). Objects as points. arXiv.

[41] Hu J., Shen L., Sun G. Squeeze-and-excitation networks. Proceedings of the IEEE Conference on Computer Vision and Pattern Recognition.

[42] Woo S., Park J., Lee J.Y., Kweon I.S. Cbam: Convolutional block attention module. Proceedings of the European Conference on Computer Vision (ECCV).

[43] Cao Y., Xu J., Lin S., Wei F., Hu H. Gcnet: Non-local networks meet squeeze-excitation networks and beyond. Proceedings of the IEEE/CVF International Conference on Computer Vision Workshops.

[44] Wang X., Girshick R., Gupta A., He K. Non-local neural networks. Proceedings of the IEEE Conference on Computer Vision and Pattern Recognition.

[45] Vaswani A., Shazeer N., Parmar N., Uszkoreit J., Jones L., Gomez A.N., Kaiser Ł., Polosukhin I. Attention is all you need. Proceedings of the NIPS’17: 31st International Conference on Neural Information Processing Systems.

[46] Carion N., Massa F., Synnaeve G., Usunier N., Kirillov A., Zagoruyko S. End-to-end object detection with transformers. Proceedings of the European Conference on Computer Vision.

[47] Han J., Ding J., Xue N., Xia G.S. Redet: A rotation-equivariant detector for aerial object detection. Proceedings of the IEEE/CVF Conference on Computer Vision and Pattern Recognition.

[48] Dai J., Qi H., Xiong Y., Li Y., Zhang G., Hu H., Wei Y. Deformable convolutional networks. Proceedings of the IEEE International Conference on Computer Vision.

[49] Yang X., Yan J. Arbitrary-oriented object detection with circular smooth label. Proceedings of the European Conference on Computer Vision.

[50] Yang X., Hou L., Zhou Y., Wang W., Yan J. Dense label encoding for boundary discontinuity free rotation detection. Proceedings of the IEEE/CVF Conference on Computer Vision and Pattern Recognition.

[51] Yu Y., Da F. Phase-shifting coder: Predicting accurate orientation in oriented object detection. Proceedings of the IEEE/CVF Conference on Computer Vision and Pattern Recognition.

[52] Liao M., Zhu Z., Shi B., Xia G.s., Bai X. Rotation-sensitive regression for oriented scene text detection. Proceedings of the IEEE Conference on Computer Vision and Pattern Recognition.

[53] Yi J., Wu P., Liu B., Huang Q., Qu H., Metaxas D. Oriented object detection in aerial images with box boundary-aware vectors. Proceedings of the IEEE/CVF Winter Conference on Applications of Computer Vision.

[54] Cheng G., Wang J., Li K., Xie X., Lang C., Yao Y., Han J. (2022). Anchor-free oriented proposal generator for object detection. IEEE Trans. Geosci. Remote Sens..

[55] Yang S., Pei Z., Zhou F., Wang G. Rotated faster R-CNN for oriented object detection in aerial images. Proceedings of the 2020 3rd International Conference on Robot Systems and Applications.

[56] Selvaraju R.R., Cogswell M., Das A., Vedantam R., Parikh D., Batra D. Grad-cam: Visual explanations from deep networks via gradient-based localization. Proceedings of the IEEE International Conference on Computer Vision.

